# A cluster randomised controlled trial of an occupational therapy intervention for residents with stroke living in UK care homes (OTCH): study protocol

**DOI:** 10.1186/1471-2377-12-52

**Published:** 2012-07-09

**Authors:** Cath M Sackley, Chris R Burton, Sandy Herron-Marx, Karen Lett, Jonathan Mant, Andrea K Roalfe, Leslie J Sharp, Bart Sheehan, Katie E Stant, Marion F Walker, Caroline L Watkins, Keith Wheatley, Jane Williams, Guiqing L Yao, Max G Feltham

**Affiliations:** 1 Faculty of Medicine and Health Sciences, University of East Anglia, Norwich, NR4 7TJ, UK; 2 School of Healthcare Sciences, Bangor University, Gwynedd, LL57 2EF, UK; 3 NHS Centre for Involvement, University of Warwick, Coventry, CV4 7AL, UK; 4 Primary Care Clinical Sciences, School of Health and Population Sciences, University of Birmingham, Birmingham, B15 2TT, UK; 5 Primary Care Unit, University of Cambridge, Cambridge, CB1 8RN, UK; 6 West Midlands Local Stroke Research Network, North Staffs Combined Healthcare NHS Trust, Stoke-On-Trent, ST4 7LH, UK; 7 Division of Mental Health & Wellbeing, Warwick Medical School, University of Warwick, Coventry, CV4 7AL, UK; 8 Division of Rehabilitation, Queen’s Medical Centre, Nottingham, NG7 2UH, UK; 9 School of Health, University of Central Lancashire, Preston, PR1 2HE, UK; 10 Birmingham Clinical Trials Unit, University of Birmingham, Birmingham, B15 2TT, UK; 11 Division of Medicine for Older People, Queen Alexandra Hospital, Portsmouth, PO6 3LY, UK; 12 Public Health, School of Health and Population Sciences, University of Birmingham, Birmingham, B15 2TT, UK

**Keywords:** Stroke, Occupational therapy, Care homes, Cluster randomised controlled trial

## Abstract

**Background:**

The occupational therapy (OT) in care homes study (OTCH) aims to investigate the effect of a targeted course of individual OT (with task training, provision of adaptive equipment, minor environmental adaptations and staff education) for stroke survivors living in care homes, compared to usual care.

**Methods/Design:**

A cluster randomised controlled trial of United Kingdom (UK) care homes (n = 90) with residents (n = 900) who have suffered a stroke or transient ischaemic attack (TIA), and who are not receiving end-of-life care. Homes will be stratified by centre and by type of care provided and randomised (50:50) using computer generated blocked randomisation within strata to receive either the OT intervention (3 months intervention from an occupational therapist) or control (usual care). Staff training on facilitating independence and mobility and the use of adaptive equipment, will be delivered to every home, with control homes receiving this after the 12 month follow-up.

Allocation will be concealed from the independent assessors, but the treating therapists, and residents will not be masked to the intervention. Measurements are taken at baseline prior to randomisation and at 3, 6 and 12 months post randomisation. The primary outcome measure is independence in self-care activities of daily living (Barthel Activities of Daily Living Index). Secondary outcome measures are mobility (Rivermead Mobility Index), mood (Geriatric Depression Scale), preference based quality of life measured from EQ-5D and costs associated with each intervention group. Quality adjusted life years (QALYs) will be derived based on the EQ-5D scores. Cost effectiveness analysis will be estimated and measured by incremental cost effectiveness ratio. Adverse events will be recorded.

**Discussion:**

This study will be the largest cluster randomised controlled trial of OT in care homes to date and will clarify the currently inconclusive literature on the efficacy of OT for stroke and TIA survivors residing in care homes.

**Trial registration:**

ISRCTN00757750

## Background

A systematic review and meta-analysis by Legg et al. [1] of nine trials (1258 participants) found that in stroke survivors, occupational therapy (OT) increased personal activities of daily living scores (i.e., standardised mean difference 0.18; 95% confidence interval 0.04 to 0.32; p = 0.01). Furthermore, for every 100 people who received OT after a stroke, 11 (95% confidence interval 7 to 30) would be spared a poor outcome (i.e., death, deterioration in personal activities of daily living or dependency on others; Odds ratio 0.67; 95% confidence interval 0.51 to 0.87; p = 0.003). A care setting where OT might be particularly beneficial is in care homes, where 20% to 40% of all people newly admitted have stroke-related disabilities as their admittance diagnosis [2,3]. Generalisation of results from community studies should be treated with caution, as the characteristics of stroke survivors resident in a care home are likely to be different to those living in their own homes. For instance, 78% of residents in a care home have cognitive impairment, 76% need some form of assistance with ambulation and 71% are incontinent [2]. These factors might affect the capacity of care home residents to engage in OT.

OT in care homes has been embraced in countries such as The Netherlands, where 93% of residents regularly receive this form of therapy [4], in contrast to the United Kingdom (UK), where as few as 3% of residents in care homes receive OT [5]. Over the last decade, the government in the UK has established a framework to assess eligibility and prioritise care needs of residents [6,7]. However, a recent audit of 112 care homes in the Midlands area of the UK found that only 6% of homes used the services of an OT at least once a week [8]. The under-utilisation of OT in this setting might be the result of staff being unaware of the role of an occupational therapist and/or how to access the services. Conversely, from a service commissioner’s perspective there is little evidence that the provision of OT services for care homes residents following a stroke is effective and/or cost-effective [8].

In the literature there is conflicting evidence on the efficacy of OT for on activities of daily life in care homes residents with stroke-related disabilities [9-11]. For instance, a study by Sackley et al. [9], which involved 249 care homes residents with mobility limitations, found that after a three month OT and physiotherapy programme there was no measureable improvements in functional independence and mobility. Although the findings suggest the therapy intervention to be ineffective, it could be argued that therapists in this study delivered interventions to maintain physical abilities of the residents rather than actively rehabilitate them. Furthermore, the therapy was applied relatively unselectively to all care home residents, rather than specifically targeted towards care homes residents with stroke-related disabilities. A cluster randomised trial, which evaluated the effect of OT compared to usual care over 3 months in 118 residents with a stroke-related disability at 12 care homes, found that residents who received OT were less likely to deteriorate in their ability to perform activities of daily living [10]. From baseline to 3 months the mean Barthel Activities of Daily Living Index (Barthel index) score had increased by 0.6 (SD = 3.9) in the intervention group, but decreased by 0.9 (SD = 2.2) in the control group. This equated to a difference between the groups of 1.5 and 95% confidence interval of -0.5 to 3.5 (allowing for a cluster design). The difference between the groups in Barthel index was maintained at 6 months (i.e., difference of 1.9 and 95% confidence interval of -0.7 to 4.4). The sample size was very small especially when taking into account the high intra class correction (ICC) of 0.37 (Barthel index at baseline), which is consistent with the ICC of 0.39 found in a subsequent pilot study of incontinence care in the same setting [11].

The findings from these studies demonstrate that even modest levels of OT may have detectable and lasting effects on morbidity and possibly mortality. It would, therefore, be appropriate to replicate the study with a larger sample to investigate the clinical impact of OT on activities of daily life in care homes residents with stroke-related disabilities.

In addition to the investigation of clinical impact of OT, a larger sample size will enable a full assessment of the economic impact that OT has on providing health care. Previous work in Canada [12] and in the UK [13] investigated the cost of OT in care homes, based on cost-consequence analyses. The Canadian study studied two types of OT and physiotherapy intensities, which compared 1 therapist to 50 bed ratio to 1 therapist to 200 bed ratio. Improvements in functional outcome measures favoured OT and physiotherapy delivered at the 1:50 ratio, which resulted in reduced direct nurse time and equated to an annual saving of 283 Canadian dollars per resident [12]. The study conducted in the UK, examined the effect of OT on levels of depression and quality of life in care homes residents [13]. It was found that, at 2005 levels, the net cost of providing the OT service was 16 British pounds per resident per week. However, it was suggested by the authors that OT might have resulted in a reduction in overall health costs [13]. Both studies suggest that providing OT to residents in a care homes incurs an initial cost to health care providers, but generates savings in the long-term through the improvement of functional outcomes of residents without stroke-related disabilities [12,13]. There is, therefore, a need to analyse the economic impact of OT on residents with stroke-related disabilities in care homes.

The purpose of this study is to conduct a large scale cluster randomised control trial to evaluate the effects of the provision of OT, which include task related training, the provision of adaptive equipment, minor environmental adaptations and staff education, compared to usual care, on activities of daily living, mobility, depression and quality of life for residents with stroke-related disabilities in care homes. Allocation of therapy cannot be concealed from the carers and/or residents so, to minimise contamination of intervention, the unit of randomisation will be the care home. Furthermore, we aim to conduct a health economic evaluation of the effect of the provision of OT services has on the health care system compared to usual care. Given the findings of previous studies on clinical [1,9-11] and health economic [12,13] impact of OT compared to usual care in the UK [5,8], it is hypothesised that the provision of OT services will have a favourable impact on activities of daily living, and reduce long-term costs to health care providers.

## Methods/Design

### Design

**Figure 1:**
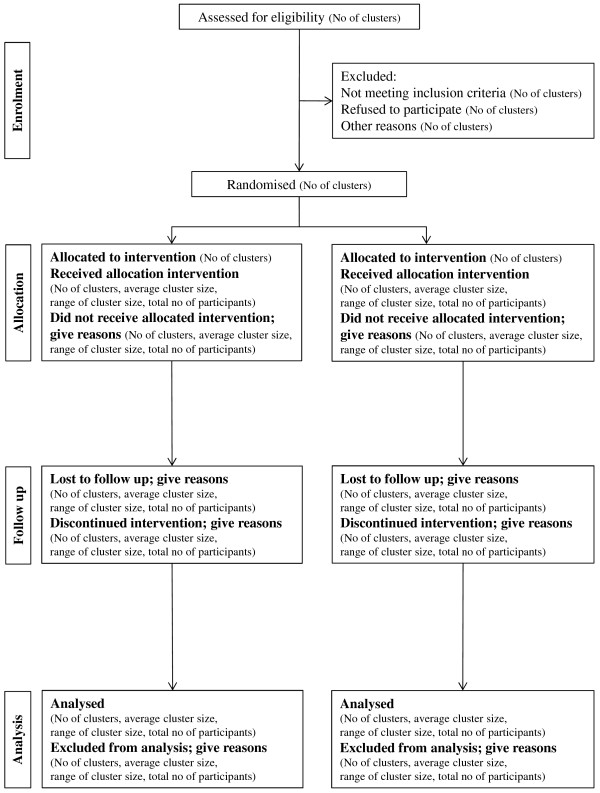
Flow diagram of the progress of clusters and individuals through phases of the trial.

This study is a pragmatic stage III cluster randomised controlled trial with health economic evaluation. The flow diagram for this trial is presented in Figure 1. and follows the CONSORT extension for cluster randomised trials [14].

### Setting

Care homes for older people in the UK from Birmingham, Bangor, Coventry, Portsmouth, Nottingham, Central Lancashire, Peninsula, Staffordshire and Wolverhampton areas. We will include all funding models of homes (i.e. private, charitable, not for profit and local authority). Homes for people with learning disabilities or drug addiction will be excluded from the study.

### Recruitment and consent

#### Care homes

Care home managers will be approached and provided with information about the study. They will be given a full oral explanation of the study, a leaflet describing OT and study information sheets. Care home managers will be asked to give written agreement for their home to participate in the study. Following this, residents will be recruited individually.

#### Residents

Care home managers will assist to identify potential participants. Participants must be resident in a care home and have a history of stroke or transient ischaemic attack (TIA). All efforts will be made to include participants with communication and cognitive impairments, because this will more accurately reflect the population characteristics. Participants will be excluded from the study if they are receiving end-of-life care (with life expectancy < 6 months). Care home members of staff will search residents’ notes to determine a diagnosis of stroke or TIA. If required, we will confirm this diagnosis with general practice records. When a potential participant is identified as being eligible for the study, they and (where appropriate) their family (outlined in the UK’s Mental Capacity Act 2005 [15]) will be approached by a senior member of the care home staff, a research network nurse or therapist, general practitioner or geriatrician, and will be asked if they are interested in participating in a research study. Prospective participants (and their family) will be given a full explanation of the study. This will include discussion of the treatment options in the trial and the manner of treatment allocation. Potential participants and their family will be given a participant information sheet, or a consultee information sheet and the UK Clinical Research Network’s 2007 [16] publication on ‘Understanding Clinical Trials’ to read. They will be given sufficient time to decide (at least 24 hours) whether they would like to join the study. This may take a few days if relatives only visit at weekends for example. Residents will then be asked to sign the consent form. If the resident is considered to be incapacitated, according to the Mental Capacity Act 2005 guidelines [15], a family member (consultee) will be approached for consent. The participant’s general practitioner will be informed, if the patient or consultee consents, in writing of their participation in the trial. Research nurses from NHS research networks will assist in the recruitment and consent processes.

### Randomisation

Homes and participants will be recruited and consented into the study before the randomisation process commences to reduce bias [17,18]. Homes will be stratified by residential care homes providing personal care and homes providing additional nursing care. Once the study co-ordinator (KES) receives confirmation from the assessors that all residents in a participating home have given their consent and completed a baseline assessment, the homes will be grouped and randomised (50:50) to receive either the OT intervention or control (usual care). The assignment of cluster to either the OT intervention or control arm of the study will be conducted by the study co-ordinator. They contact the managers of the homes directly and make arrangements for the occupational therapist to visit the care home to commence the intervention. The allocation sequence will be generated by a statistician (AKR) using blocked randomisation within strata (centre and type of home; residential, nursing) at the Primary Care Clinical Research and Trials Unit at the University of Birmingham, independent from the research team. The details of the sequence will be unknown to any of the assessors or to the study co-ordinator. Allocation will be concealed from the independent assessors, but it is not possible to mask the treating therapists or residents from the intervention. The success of blinding will be evaluated at each follow-up stage by asking the assessors to record if the allocation has been revealed to them.

### Intervention

We developed an OT intervention package for residents in a care home using evidence and consensus from a previous study [19]. The OT intervention will be provided by qualified occupational therapists and assistants and will be targeted towards improving independence in activities of daily living, such as feeding, dressing, toileting, bathing, transferring and mobilising. Adaptive equipment will be provided as part of the study and will include personal items, such as adapted cutlery and walking aids. Furthermore, adaptations to the individual's environment might need to be made, such as chair raisers, bed levers, raised toilet seats or grab rails. When adaptive equipment or adaptations to the environment have been provided to the resident, the occupational therapist will demonstrate to them and the care staff how to use the equipment effectively, whilst adhering to safety regulations. The frequency and duration of the OT will be dependent on the resident and therapist’s agreed goals (within the framework of the home). In the pilot study, the number of face-to-face sessions ranged from 1 to 25 per resident over a three-month period (median time = 8.5 hours and mean time = 4.7 hours), dependent on the individual needs of the resident [19]. The OT intervention will follow a ‘client centred approach’ [20] and will include a continuous process of assessment, treatment and reassessment. In line with current evidence on effective treatment, the OT intervention will adopt a task-specific training approach [21]. Treatment logs have been developed in which the dose and focus of OT intervention can be documented. This will allow for accurate costing for health care services to residents in care homes to be calculated. Care homes in the control arm of the study will continue to provide their usual care to residents (i.e. continue providing (or not) any therapy as they would do usually).

### Training for care home staff

Specific training will be given for staff involved in the care of the residents receiving active therapy. In addition, all intervention homes will be offered a group training session on the key principles of OT by the occupational therapist. A half-day group training session for care home staff has been developed and summarised, on the facilitation of independent daily living activities and mobility of residents [22].

Care homes in the control arm will receive similar training, but only after the 12 month follow-up assessment has been completed. It is anticipated that delivering the training to the control group will facilitate compliance and reduce loss to follow-up in the control group. This, in turn, will reduce the potential bias during the follow-up period [19].

### Outcome measures

#### Demographic data

Information recorded by a member of staff from the care home about the resident will include age, gender, current medication intake and date, type and side of stroke. Furthermore, at baseline a trained assessor will administer the Sheffield Screening Test for Acquired Language Disorders, to assess receptive/expressive aphasia [23], and the mini mental state examination to assess cognitive function [24]. Finally, data on resource use will be recorded through logs and collected from health and social care services to conduct a health economic evaluation for both interventions delivered during this study.

#### Primary outcome measure

The primary outcome measure is the Barthel Activity of Daily Living Index [25], which is a commonly used measure of independent self-care in people who survived a stroke [26,27]. Furthermore, the Barthel index assesses specific aspects of self care targeted by the therapy. A change of two points on this scale is widely accepted as being clinically meaningful [28]. The Barthel index can be completed by the resident or with assistance from a member of staff.

#### Secondary outcome measures

Secondary outcome measures will assess mobility, mood, safety, quality of life and costs. Mobility will be assessed with the Rivermead mobility index [29], which is a 15-item measurement of functional mobility. The geriatric depression scale [30] will be administered to measure mood. The full 30-item version will be used as standard, but in cases where residents are unable to self complete or follow and interview the informant version will be filled out. Quality of life is measured by the EQ-5D [31], which is a well established measure for evaluating patients preference. Costs will be estimated based on resource usage log and weighted by unit costs. This will include visits to hospital and include in- and out-patient appointments, allied health professionals time, general practitioner visits, provision of care in a care home, adaptive equipment, minor environmental adaptations and staff education. Unit costs will be obtained from the national health and social care services reference costs and personal social services research unit [32]. Additionally, any adverse events will be recorded in participant logs and will include a fall or equipment failure leading to an injury requiring a visit to a hospital or general practitioner.

#### Assessment schedule

An overview of the assessment schedule is given in Table 1. Baseline assessments will be conducted prior to randomisation to reduce possible recruitment bias repetition [33]. The primary measurement point will be at 3 months after randomisation and follow-up assessments will be conducted at 6 and 12 months prior to randomisation. Data will be collected from participants where they are currently residing. If a participant moves to another home between assessments, then the assessors will attempt to collect follow-up data.

**Table 1:** Proposed assessment schedule

**Assessment**	**Time of administration**			
	Baseline	3 months	6 months	12 months
Demographics	✓			
Sheffield Screening Test*	✓			
Mini Mental State Examination	✓			
Barthel Activity of Daily Living Index	✓	✓	✓	✓
Rivermead Mobility Index	✓	✓	✓	✓
Geriatric Depression Scale	✓	✓	✓	✓
EQ-5D	✓	✓	✓	✓
Resource Use Log		✓	✓	✓
Adverse Event Log		✓	✓	✓

*Full name of the test is Sheffield Screening Test for Acquired Language Disorders.

### Sample size

A sample of 330 residents in each arm will be sufficient to identify a clinically meaningful 2-point change in the Barthel index. This estimate is based on a standard deviation of 3.7 [10], 90% power, 5% level of significance and an ICC of 0.4 [10,11]. Given that the trial will be a cluster randomised trial, it is predicted that 33 homes with 10 residents in each will be required in each arm of the study. Based on the attrition rate of 26% from a previous study [10], it is predicted that 45 homes with 10 residents in each will be required in each arm of the study (i.e., 900 residents in total).

### Statistical analysis

Baseline characteristics of participants will be tabulated by treatment arm. Items will include demographic details of residents, past medical history, questionnaires; and breakdown of the number of care homes and cluster size.

#### Primary analysis

The primary outcome is the mean Barthel index score at the 3 month follow-up. A mixed model analysis will be used to compare Barthel index scores between the intervention and control groups. The primary analysis will be adjusted by care home (as a random factor), baseline Barthel index score, and stratification factors: centre and type of care home (nursing, residential). Participants that die before their follow-up date will be given a Barthel index score of zero at all subsequent follow-ups. In addition, participants will be categorised into 3 outcome groups based on an individual’s change in Barthel index score at 3 months from baseline (below 0 or death = ‘poor’, 0 to 1=’moderate’, 2 and above=’good’). A non-linear mixed effects model will be used to compare this ordinal outcome between the groups. Adjustments will be made for care home (random), centre and type of care home (fixed).

#### Secondary analysis

To identify any longer term effects, a repeated measures mixed model analysis of the primary outcome will be undertaken, comparing groups across all time points. The analysis will include adjustment for care home (as a random factor), baseline Barthel index score, and stratification factors: centre and type of care home. Similar analyses will be performed for mobility, mood and quality of life.

The number of falls will be compared using a Poisson or negative binomial model, with adjustments for care home, centre and type of care home as previously described. A sensitivity analysis of the outcomes will be performed to examine the potential effect of missing data. This will include best case, worst case and multiple imputation methods.

Analysis will be by intention to treat, whereby residents will be analysed according to the intervention to which they are randomised, regardless of whether they comply with the treatment. All participants irrespective of when they die, withdraw or are lost will be included in the arm to which they were randomised, including those that die after consent but prior to randomisation. Those participants that move home will be analysed by the home they were originally randomised to. Analyses will be performed using Stata (StataCorp,. Texas, USA) and SAS software (SAS Institute Inc., Cary, NC).

#### Health economic evaluation

Outcomes from health economic evaluation analyses will establish cost effectiveness of a treatment expressed as incremental cost per quality-adjusted life-years (QALYs) gained. Generalised linear models will be used to investigate differences between interventions for QALYs and costs. Bootstrap methods will be adopted to produce incremental cost effectiveness ratio and associated confidence intervals. Cost-effectiveness acceptability curves will be generated to reflect the probability that a treatment will be cost effective given a society’s willingness to pay in terms of price per QALY gained.

### Governance

#### Ethical approval

Favourable ethical opinion for this study was obtained from the National Research Ethics Service, Coventry Research Ethics Committee (Reference: 09/H1210/88). The study was granted an International Standard Randomised Controlled Trial Number (00757750).

#### Trial management

A trial steering group has been established to monitor the governance of the study consisting of the main research team, an independent chair, a geriatrician, an occupational therapist, a physiotherapist with expertise in rehabilitation research a patient representative, and a representative from the National Institute for Health Research. A data monitoring committee has been established with an independent geriatrician as chair, a statistician and an occupational therapist. During the development of this protocol, a patient representative was consulted.

#### Service user involvement

Service user (and carer) involvement will be incorporated at all levels of this study. Service user (and carer) involvement will not be a ‘stand-alone’ activity but rather an integral part of all aspects of this study. Service users (and carers) will be directly involved as research ‘partners’ and not just as ‘data providers’. All support for service users (and carers) involvement will be provided by the Stroke Research Network members who have expertise in training and supporting service users (and carers) for involvement in National Health Service research, service evaluation and development.

## Discussion

The methodological limitations and considerations of cluster randomised trials are well reported [34] and have been addressed in the current study protocol. Furthermore, the choice of clustering design to reduce intervention contamination was a key component of the development of this trial. Previous cluster randomised trials demonstrated that a relatively small level of OT may have detectable and relatively long-lasting effects on morbidity and possibly mortality in care homes residents with stroke-related disabilities [10,11]. These studies enrolled small numbers of clusters and participants and were conducted in specific areas of the UK, which reduces the generalisability of the findings to residents in care homes more widely. The current study will be the largest cluster randomised control trial of OT in care homes to date. It will provide clinically relevant information on the effect of OT services on activities of daily life compared to usual care. Furthermore, it aims to establish the effects of OT services on aspects such as mobility, depression and quality of life, which are major challenges experienced by many residents [2]. Finally, this study will evaluate the health economic impact of OT services compared to usual care. In combination, the findings of this large scale cluster randomised controlled trial will assist in the formulation of UK guidelines and policies on the provision of OT services for residents with stroke-related disabilities in care homes.

## Abbreviations

OTCH, Occupational therapy in care homes; OT, Occupational therapy; UK, United Kingdom; QALY, Quality adjusted life years; ICC, Intra class correction; TIA, Transient ischaemic attack.

## Competing interests

The authors declare that they have no competing interests.

## Authors’ contributions

CMS was the Chief investigator. MGF drafted the manuscript. KES is the main trial coordinator and will make a substantial contribution to the acquirement and analysis of data. AKR provided statistical support. GLY provided expertise on health economic evaluation. CRB, SHM, KL, JM, LJS, BS, MFW, CLW, KW and JW contributed significantly towards the development of the study protocol. All authors read and approved the final manuscript.

## Department of Health Disclaimer

The views and opinions expressed therein are those of the authors and do not necessarily reflect those of the Health Technology Assessment programme, National Institute for Health Research, National Health Service or the Department of Health.

## Trial funding

National Institute for Health Research Health Technology Assessment Programme (project number 08/14/30).

## Pre-publication history

The pre-publication history for this paper can be accessed here:

http://www.biomedcentral.com/1471-2377/12/52/prepub
